# Laparoscopic Ladd surgery in adult with chronic intestinal obstruction due to midgut malrotation

**DOI:** 10.1093/jscr/rjag082

**Published:** 2026-05-29

**Authors:** Benjamin Thorpe Plaza, Wilson Darío Rodríguez Velandia, Purificación Parada González

**Affiliations:** General Surgery Department, Hospital Clinico Universitario de Santiago de Compostela, Santiago de Compostela, Spain; General Surgery Department, Hospital Clinico Universitario de Santiago de Compostela, Santiago de Compostela, Spain; General Surgery Department, Hospital Clinico Universitario de Santiago de Compostela, Santiago de Compostela, Spain

**Keywords:** intestinal malrotation, Ladd surgery, midgut volvulus, minimally invasive surgery

## Abstract

Symptomatic congenital intestinal malrotation during adulthood is a rare condition usually presented as a chronic clinical condition superimposable to an upper intestinal obstruction. Symptoms tend to be nonspecific, varying from intermittent episodes of vomiting, food intolerance, and weight loss, leading to a delayed diagnosis worsening the patient’s functional reserve. High clinical suspicion leads to the request of the request of requesting adequate diagnostic imaging, with computerized tomography being the imaging of choice. While Ladd surgery is the standard of care, the approach is controversial with a tendency for minimal invasive surgery due to faster postoperative recovery, better pain management, and shorter hospital stays. A case of female patient in her 80s is presented with 6 months symptoms of vomiting and weight loss. Computed tomography scan diagnosed an upper intestinal obstruction due to intestinal malrotation. Intraoperative findings showcased a duodenojejunal junction stenosis due to the presence of Ladd bands. Ladd surgery was performed without concomitant appendicectomy.

## Introduction

Congenital intestinal malrotation (CIM) refers to an abnormal fixation of the midgut in the abdominal cavity due to a failure during the rotation of the intestine around the superior mesenteric artery (SMA) during the 4th to 12th weeks of gestation [1–3]. While in children the incidence is around 1:500 live births [2], in adults it is extremely rare, and its true incidence is not accurately known, with some series describing rates of 0.2% to 0.5% [2–5]. Even though CIM generally remains asymptomatic during adulthood, it can potentially lead to life-threatening clinical scenarios such as acute duodenal obstruction, midgut volvulus, bowel strangulation, and ischemia [4, 6, 7].

The typical clinical presentation in adults generally manifests as chronic, non-specific intermittent abdominal pain followed by food intolerance, weight loss, and malnutrition [4, 5, 8, 9]. The nonspecific clinical symptoms, often seen in other causes of intestinal obstruction, make the diagnosis challenging, requiring a high clinical suspicion as well as specific diagnostic imaging showcasing an obstruction at the duodenojejunal junction in the context of intestinal malrotation [3–5]. While upper gastrointestinal (UGI) series and Doppler ultrasound of the SMA are the standard modalities in paediatric patients, in adults, CT scanning is the modality of choice, achieving success rates as high as 97% [5, 10, 11].

## Case presentation

A female patient in her early 80s, with a previous surgical history of laparoscopic cholecystectomy and umbilical hernia repair, presented to the outpatient clinic due to a 6-month constitutional syndrome characterized by a 15-kg weight loss, abdominal distention, epigastric pain, a sensation of gastric heaviness, decreased appetite together with nausea, and one to two episodes of vomiting per week. Otherwise, she presented normal bowel movements, with a slight tendency towards constipation. During anamnesis, the patient remained clinically stable with normal vital signs. The abdominal physical examination was benign, with mild tenderness in the epigastric region but no further signs of pain or peritonitis.

**Figure 1:**
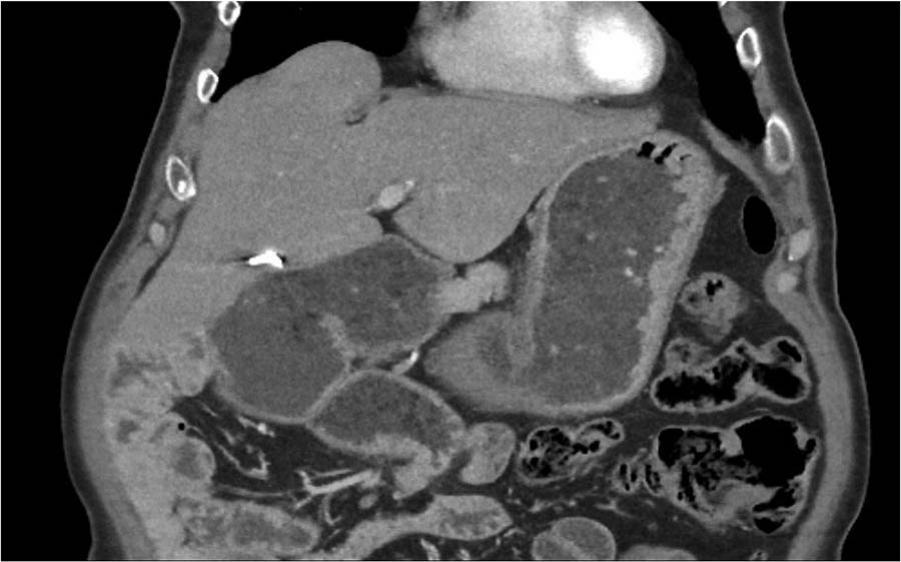
CT imaging with signs of intestinal malrotation associating important gastric and duodenal dilatation with a subsequent change in calibre at the duodenojejunal junction.

An upper endoscopy was performed, describing significant gastric and duodenal dilation up to the inferior duodenal flexure, where an abrupt angulation was present. Passage was only possible with a paediatric endoscope, showcasing a normalization of the jejunal lumen calibre once the duodenal stenosis was overcome. Studies were completed with a computed tomography (CT) scan, describing signs of intestinal malrotation with marked gastric and duodenal dilation up to a change in calibre at the duodenojejunal junction (Fig. 1). Laboratory parameters were not remarkable, with mild leucocytosis, C-reactive protein elevation, and normal procalcitonin levels. Lactate was within normal range values.

After the diagnosis of UGI obstruction, suspected midgut volvulus, and CIM, parenteral nutrition was initiated, and emergency surgery was scheduled. Previous laparoscopy had not revealed intestinal malrotation, and the patient’s clinical presentation was rather chronic than acute, which favoured the diagnosis of upper GI obstruction rather than midgut volvulus. For these reasons, an exploratory laparoscopy was performed to clarify the definitive diagnosis.

Intraoperative findings showcased significant duodenal dilation, reaching the second duodenal knee, towards the duodenojejunal junction, where a severe stenosis was evident. The angle of Treitz was not fixed and was located on the right side of the abdominal cavity. The duodenal passage was compromised at the duodenojejunal junction by the presence of fibrotic peritoneal tissue compatible with Ladd bands. The duodenum itself was included in a peritoneal sac, which presented adhesions to the surgical site of the previous cholecystectomy. No signs of duodenal volvulus or intestinal ischemia were evident during the operation.

In response to the intraoperative findings, a modified Ladd procedure was performed. Ladd bands were divided, widening the midgut mesentery. Laparoscopic adhesiolysis of the previous gallbladder surgical bed and release of the peritoneal sac enveloping the duodenum were performed, achieving good mobilization and verticalization of the duodenum and reinstating duodenal peristalsis, which was immediately evident. Appendectomy was not performed to avoid converting from a clean to a clean-contaminated surgery and to minimize associated morbidity. The small bowel was placed on the right side of the abdomen and the colon on the left side, with no subsequent abdominal fixation.

**Figure 2:**
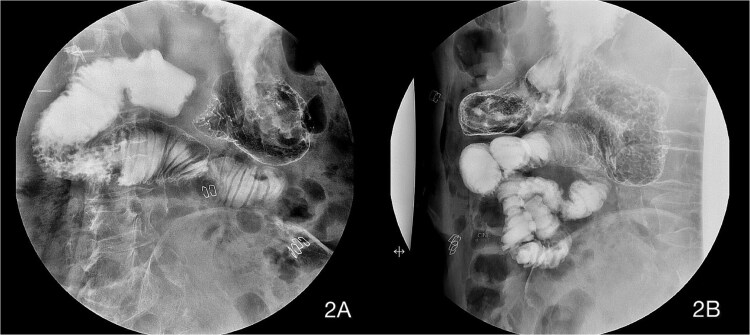
Upper gastrointestinal imaging (UGI) contrast series on the third postoperative day showing an absence of stenosis at the duodenojejunal junction with good contrast passage. A residual but less pronounced dilation of the duodenum was still present, but with good peristalsis.

**Figure 3:**
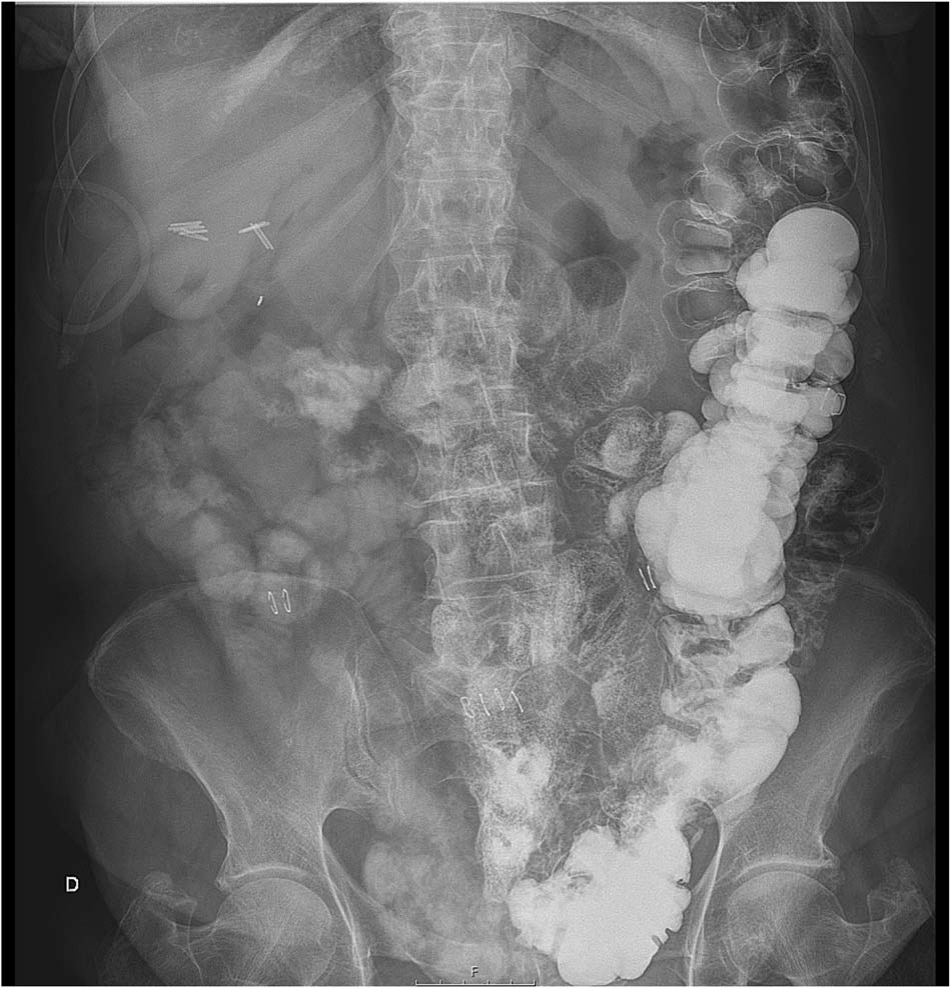
Abdominal radiography on the third postoperative day describing oral contrast on the colon which is entirely on the left side of the abdomen.

Before starting enteral nutrition, an UGI contrast study was performed on the third postoperative day, showing an absence of stenosis at the duodenojejunal junction with good contrast passage, corroborating the resolution of the duodenal obstruction. A residual but less pronounced dilation of the duodenum was still present, but with good peristalsis (Fig. 2a and b ). On the same afternoon, an abdominal radiograph showed contrast in the colon, which was entirely located on the left side of the abdomen (Fig. 3), and enteral feeding was reinstated.

The patient’s postoperative recovery was uneventful. Progressive oral tolerance was re-established, and parenteral nutrition was withdrawn. Clinical symptoms of intestinal obstruction disappeared, as well as the colic epigastric pain. The patient was discharged from the hospital on the sixth postoperative day and remained asymptomatic after 6 months of follow-up, being discharged from outpatient consultation.

## Discussion

Both in acute and chronic settings, symptomatic CIM is treated surgically with the aim of resolving the anatomical obstruction [5]. When clinically feasible, elective surgery is preferred over urgent intervention due to better outcomes, including shorter hospital stay and lower mortality rates [5, 8] . Despite emerging surgical techniques, Ladd’s procedure has proven to be a safe and effective operation with low recurrence rates [6–8], remaining the standard of care for CIM [5, 8, 9].

Ladd surgery is a standardized five-step procedure, including division of Ladd’s bands, detorsion of the rotated midgut, widening of the mesentery, placement of the small bowel on the right side and the cecum on the left side of the abdomen, and prophylactic appendectomy [5–7]. Since first described in 1932, modifications have been introduced based on intraoperative findings, individual patient characteristics, and appendix management [12, 13]. Amongst these techniques, intestinal fixation manoeuvres (duodenopexy and cecopexy) remain controversial due to the increased risk of internal hernias. Kareem’s procedure, although promising, lacks sufficient long-term outcome data [2].

The benefits of performing appendectomy during Ladd’s procedure remain controversial [13]. Recent paediatric studies show that between 70.2% [12] and 90% [13] of surgeons still perform appendectomy due to concerns regarding the future misdiagnosis of appendicitis. Nevertheless, widespread access to diagnostic imaging in modern hospitals [14], along with accurate documentation of CIM, should reduce this risk. Preservation of the appendix, due to its role in gut microbiota and intestinal immune function, supports avoiding prophylactic appendectomy [13, 15–18]. Given that peak appendicitis incidence occurs between 20 and 30 years of age, appendectomy was omitted in our elderly patient to avoid unnecessary morbidity.

Regarding the surgical approach, despite the increasing adoption of minimally invasive surgery (MIS), most procedures reported in the literature are still performed using an open approach [5–7]. Concerns regarding inadequate mesenteric widening and recurrent volvulus are the main reasons for this preference [1]. However, recent studies show that open surgery is associated with higher rates of postoperative ileus, nausea, vomiting, and longer hospital stay, compared to MIS, which demonstrates lower rates of adhesive small bowel obstruction and readmission [1, 6, 7]. Smaller incisions and regional anaesthesia techniques reduce postoperative pain and promote faster recovery [1, 6]. Ultimately, the choice of approach depends on surgeon experience, clinical presentation, and patient stability.

When comparing MIS techniques, no significant differences in postoperative recovery are observed between laparoscopic and robotic approaches. Robotic surgery offers improved visualization [6], potentially reducing conversion rates. However, costs are approximately three times higher than laparoscopy [1, 6], and limited availability necessitates careful patient selection to optimize resource utilization.
